# Molecular characterisation of second-line drug resistance among drug resistant tuberculosis patients tested in Uganda: a two and a half-year’s review

**DOI:** 10.1186/s12879-022-07339-w

**Published:** 2022-04-11

**Authors:** Dennis Mujuni, Dianah Linda Kasemire, Ivan Ibanda, Joel Kabugo, Andrew Nsawotebba, Jody E. Phelan, Robert Kaos Majwala, Didas Tugumisirize, Abdunoor Nyombi, Beatrice Orena, Irene Turyahabwe, Henry Byabajungu, Diana Nadunga, Kenneth Musisi, Moses Lutakoome Joloba, Willy Ssengooba

**Affiliations:** 1 Makerere University, College of Health Sciences, Kampala, Uganda; 2 World Health Organisation Supranational Reference Laboratory, Uganda National TB Reference Laboratory, Kampala, Uganda; 3 Department of Pharmacology and Toxicology, School of Pharmacy, Kampala International University, Kampala, Uganda; 4 National Health Laboratory and Diagnostic Services, Kampala, Uganda; 5 Faculty of Infectious & Tropical Diseases, London School of Hygiene & Tropical Medicine, London, UK; 6 United States Agency for International Development, Defeat TB Project, Kampala, Uganda; 7 National Tuberculosis and Leprosy Control Programme, Ministry of Health, Kampala, Uganda; 8 World Health Organisation EPI Laboratory, Uganda Virus Research Institute, Entebbe, Uganda; 9 Department of Medical Microbiology, School of Biomedical Sciences, Makerere University, Kampala, Uganda; 10 Makerere University Lung Institute, Makerere University College of Health Sciences, Kampala, Uganda

**Keywords:** *Mycobacterium tuberculosis*, Drug-resistant tuberculosis, Drug susceptibility testing, Second line probe assay

## Abstract

**Background:**

Second-line drug resistance (SLD) among tuberculosis (TB) patients is a serious emerging challenge towards global control of the disease. We characterized SLD-resistance conferring-mutations among TB patients with rifampicin and/or isoniazid (RIF and/or INH) drug-resistance tested at the Uganda National TB Reference Laboratory (NTRL) between June 2017 and December 2019.

**Methods:**

This was a descriptive cross-sectional secondary data analysis of 20,508 M*. tuberculosis* isolates of new and previously treated patients’ resistant to RIF and/or INH. DNA strips with valid results to characterise the SLD resistance using the commercial Line Probe Assay Genotype *MTBDRsl* Version 2.0 Assay (Hain Life Science, Nehren, Germany) were reviewed. Data were analysed with STATAv15 using cross-tabulation for frequency and proportions of known resistance-conferring mutations to injectable agents (IA) and fluoroquinolones (FQ).

**Results:**

Among the eligible participants, 12,993/20,508 (63.4%) were male and median (IQR) age 32 (24–43). A total of 576/20,508 (2.8%) of the *M. tuberculosis* isolates from participants had resistance to RIF and/or INH. These included; 102/576 (17.7%) single drug-resistant and 474/576 (82.3%) multidrug-resistant (MDR) strains. Only 102 patients had test results for FQ of whom 70/102 (68.6%) and 01/102 (0.98%) had resistance-conferring mutations in the *gyrA* locus and *gyrB* locus respectively. Among patients with FQ resistance, *gyrAD94G* 42.6% (30.0–55.9) and *gyrA A90V* 41.1% (28.6–54.3) mutations were most observed. Only one mutation, E540D was detected in the *gyrB* locus. A total of 26 patients had resistance-conferring mutations to IA in whom, 20/26 77.0% (56.4–91.0) had A1401G mutation in the *rrs* gene locus.

**Conclusions:**

Our study reveals a high proportion of mutations known to confer high-level fluoroquinolone drug-resistance among patients with rifampicin and/or isoniazid drug resistance. Utilizing routinely generated laboratory data from existing molecular diagnostic methods may aid real-time surveillance of emerging tuberculosis drug-resistance in resource-limited settings.

## Background

Tuberculosis (TB) remains one of the world’s deadliest infectious diseases, with an average of 10 million infections and 1 million deaths annually, even when it remains curable [1, 2]. A tota 88,000 new TB cases, which translates into 200/100,000 population were reported in 2019 in Uganda[3, 4]. In the same year, a total of 1500 new Multidrug resistant/rifampicin resistant (MDR/RR) TB cases were estimated to be reported. Important to note is that only 559 (37%) of the estimated MDR/RR-TB cases were diagnosed and notified to the National TB and Leprosy Program (NTLP) in 2019 [5] of whom 180 (12%) were previously treated [3, 4].

Uganda is among the 30 High TB/HIV burden countries [1, 2]. Despite the increase in TB notification, the progress to address detection and treatment gaps is still slow and large gaps remain [4, 6, 7]. Amidst all this, drug-resistant TB (DR-TB) remains the most critical challenge facing global TB control. The prevalence of MDR-TB in Uganda in 2019 was estimated to be 1% (0.93–1.2) among newly diagnosed TB cases and 12% (6.5–19) among previously-treated TB cases [4]. Availing important drug resistance data at periodic intervals in the wait for prevalence and drug resistance surveys has a key role to play towards DR-TB control.

Uganda has a comprehensive TB diagnostic network that consists of 100 laboratories/transport hubs and ~ 251 GeneXpert sites but these only diagnose rifampicin resistance. Molbio Truenat MTB/RIF (TrueNat) assay has also been proposed and introduced to increase TB detection at primary health centre level, where power supply and air conditioning is lacking [8]. In Uganda, the National TB Reference Laboratory (NTRL), which serves the entire country, employs the traditional methods of drug susceptibilty testing (DST) such as solid and liquid phenotypic testing for fluoroquinolones (FQ) and injectable agents (IA). The conventional DST methods are tedious, with a long turn-around time and technical challenges, more so with critical concentrations for new anti-TB drugs. This is partly addressed by the use of rapid diagnostics such as the Line Probe Assays (LPA) for genotypic DST. The second-line probe assay test strip allows for the detection of specific mutations in the quinolone resistance-determining region (QRDR) and IA [9]. Specific mutations in the QRDR confer drug resistance to FQ (levofloxacin LXF, ofloxacin OFX, moxifloxacin MXF), whereas those in the *rrs* gene confer drug resistance to IA (amikacin AMK, kanamycin KAN, and capreomycin CAP) [10]. Increased drug resistance levels have in the recent past been linked to poor treatment outcomes [11]. Of the 384 patients that started on second line treatment in the MDR-TB Cohort of 2017 in Uganda, 74% were successfully treated [4]. This raises concerns about the eventual total costs after failed treatments and repeated diagnosis eventually engineering drug resistance. There is an urgent need for the protection of novel drugs through timely surveillance of resistance outbreaks.

Besides offering rapid DST beyond first-line anti-TB drugs, LPAs additionally provide data that can enhance drug resistance surveillance in the absence of drug resistance surveys. This would guide TB control, surveillance and management efforts from disease control programs. This study therefore aimed at offering timely findings on the molecular characterisation of second line drug resistance among patients resistant to isoniazid and/or rifampicin, using the second-line LPA since its routine use in Uganda.

## Methods

### Study design and setting

This was a descriptive cross-sectional secondary data analysis involving extraction and review of data from samples of TB patients received between June 2017 and December 2019 at the NTRL in Kampala. Data extraction and review were performed on 20,508 isolate entries of TB patients for DST. The NTRL is a Biosafety Level (BSL) 3 Laboratory that is fully furnished to manipulate TB cultures and specimens [12].

### Study population

This study included sputum specimens from patients having either pulmonary or extra-pulmonary TB including samples from patients with history of previous TB treatment who were referred to NTRL for DST from testing centers country-wide. For analysis we included results of patients who had RR-TB detected on GeneXpert/Ultra and patients with rifampicin and/or isoniazid resistance detected using LPA and/or Mycobacteria Growth Indicator Tube (MGIT) SIRE DST kit.

### Sample processing, culture and drug susceptibility testing

**Fig. 1:**
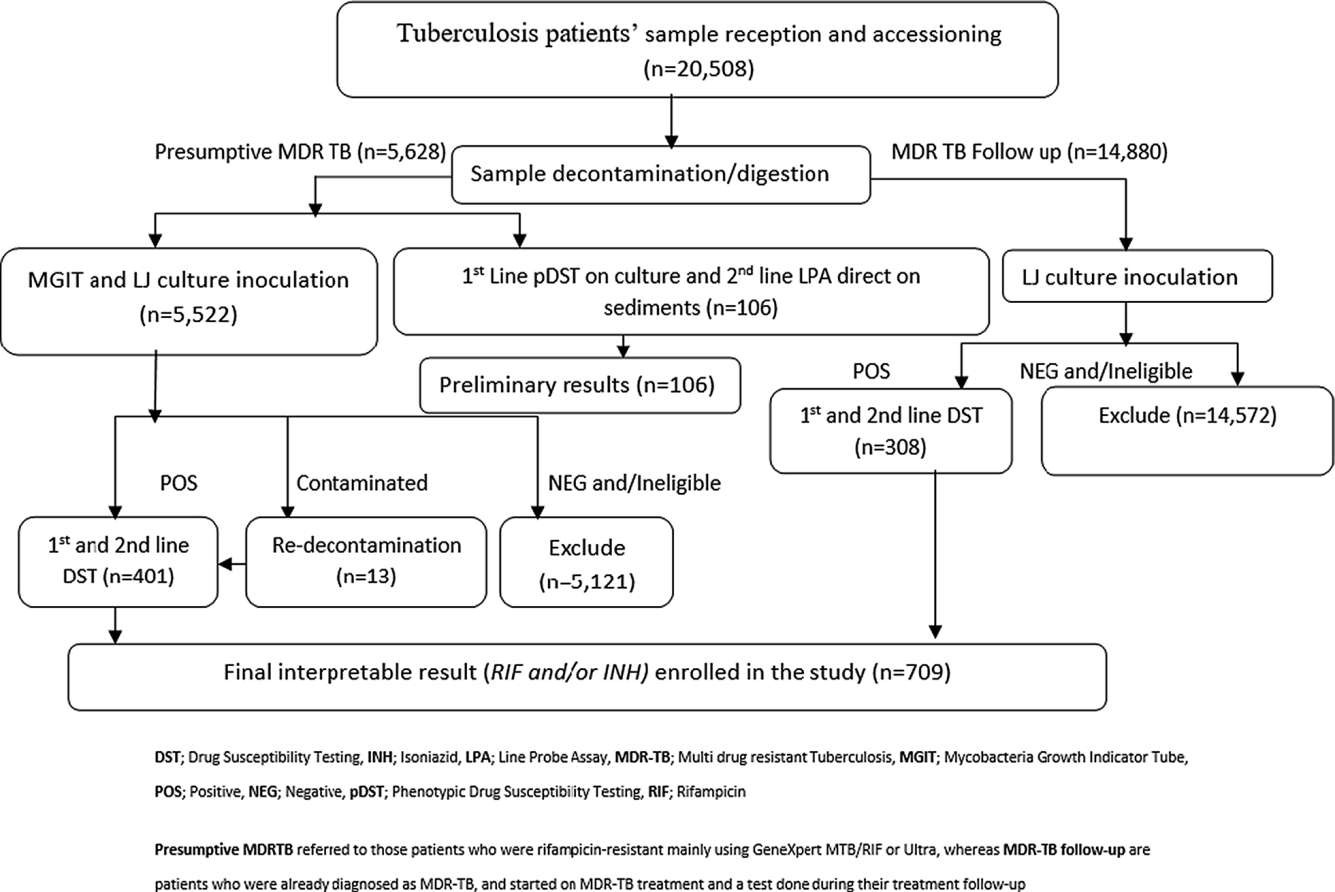
Flow chart for sample processing and enrolment into the study

Using the Uganda National TB specimen transport system, all samples were transited to NTRL under a cold chain. Upon receipt, samples were accessioned for satisfaction of the minimum sample acceptance criteria prior to sample processing. Samples were processed using N-Acetyl-L-Cysteine-Sodium hydroxide (NALC-NaOH; 1.5% NaOH final concentration). The specimens were then inoculated on both Lowenstein Jensen (LJ) media and MGIT. MGIT DST for the first and SLDs was performed according to standard operating procedure [13]*.* DNA extraction for second line LPA DST was performed in the BSL3 using the GenoLyse® kit; Genotype MTBDR*sl V2.0* (Hain Lifescience, Nehren, Germany) assay (direct) on the processed sediments or (indirect) on the respective positive cultures of the eligible participants and results interpreted according to standard procedures [9, 14]. All procedures were done according to standard procedures [9, 14], Fig. 1.

### Laboratory quality control

Data quality control measures were taken using the NTRL Laboratory Information Systems to ensure that non-repetitive *M. tuberculosis* clinical patient isolates were considered for the study. The reviewed samples had acceptable internal quality control (IQC) results tested along each run performed. Comparability testing was performed using the second line DST kit on the MGIT960 System (Becton Dickinson, Franklin Lakes, NJ). In fulfillment of External Quality Assurance of the test, LPA testing was also performed on well characterized proficiency testing strains from the Supranational Reference Laboratory, Antwerp, Belgium, provided annually. The test reagents and consumables used for testing clinical samples also had records of LOT-to-LOT testing to ascertain performance. A competent reviewer also reviewed the mutation interpretations after retrieval of the data considered for the study period, all following initial review performed by other independent personnel as per the required (ISO 15189:2012) international standards [15] for medical and testing laboratories. Fading of the LPA bands was minimized by adding cello-tape onto the strips and sticking them against the worksheet, which was then kept within a sheet protector. In this way, the bands were visible to the naked eye for all, except 10 strips.

### Statistical analysis

The input mutations were first sorted, cleaned and organized in Microsoft Excel 2016. The data, consisting of other sociodemographic data such as Age, Gender and History of TB treatment was then imported into STATAv15 for analysis using the cross-tabulation technique with descriptive statistical tests using frequencies and proportions. Confidence Intervals at a 5% level of significance were computed for some of the proportions to make inferences. The results were then presented as frequencies in the form of a table and graph.

## Results

Overall, 20,508 entries of new and previously treated patients were screened for eligibility, largely consisting of males 12,993/20,508 (63.4%). The median age (Interquartile range; IQR) of the participants was 32 (24–43) and a total of 14,880/20,508 (72.6%) were previously treated for TB, Table 1.

**Table 1:** Sociodemographic characteristics of the patients

Variable (N = 20,508)	n (%)
Age	
Median (IQR)	32 (24–43)
Age groups	
< 35 years	11,147 (54.3)
≥ 35 years	9361 (45.7)
Sex	
Female	7515 (36.6)
Male	12,993 (63.4)
History of TB treatment	
Previously treated	14,880 (72.6)
New case	2871 (14.0)
Regimen history unknown	10 (0.1)
Not provided	2747 (13.3)

*N* Sample size, *n* Frequency, *%* Percentage, *IQR* Interquartile Range

A total of 709/20,508 (3.45%) patients had results of first line DST to rifampicin and/or isoniazid, of whom 576/709 (81.2%) were classified as rifampicin and/or isoniazid resistant. A total of 102/576(17.7%) was classified as single drug resistant (SDR) of whom 92/102 (90.2%) [82.7–95.2] and 10/102 (9.8%) [4.8–17.3] were rifampicin mono-resistant and isoniazid mono-resistant respectively, and 474/576 (82.3%) were MDR, Table 2.

**Table 2:** Overall drug resistance per first-line Anti-TB drug tested

First Line DR (N = 709)	Test result, n (%)				
	Resistant*	Susceptible	Indeterminate	Invalid	Inferred
RIF	566 (79.8)	101 (14.2)	42 (5.9)	0 (0.0)	0 (0.0)
INH	486 (68.3)	182 (25.7)	43 (6.1)	0 (0.0)	0 (0.0)

*N* Sample size, *n* Frequency, *%* Percentage, *RIF* Rifampicin, *INH* Isoniazid, *=include MDR

### Distribution and characterization of second line drug resistance-conferring mutations

Only 68/576 (11.8%) of the patients had resistance to any of the SLD of whom 42/68 (61.8%) and 12/68 (17.6%) were classified with resistance to fluoroquinolones only and injectable agents only, respectively. Among the patients with SLD resistance results, 54/68 (79.4%) were either FQ/IA resistant, while 14/68 (20.6%) were resistant to both FQ and IA. A total of 249/20508 (1.2%) patients had indeterminate results for either one or both of the SLD. Of these, 31/249 (12.4%) were IA indeterminate only whereas 1/249 (0.4%) was FQ indeterminate only. A total of 217/249 (87.1%) was FQ and IA indeterminate. There were no cases of inferred SLD resistance with one invalid result in our study.

The distribution of the SLD resistance across all patient variables was highest amongst the patients of < 35 years of age, and males dominated in each of them. Previously treated TB patients were the majority in all categories of SLD resistance. We observed that history of TB treatment was not a subject of vulnerability for any type of drug resistance among our study participants. The distribution of SLD resistance is presented in Table 3.

**Table 3:** Distribution of second-line drug resistance amongst the patient variables

Variable	% Proportion (95% Confidence interval)			
	FQ Only (n = 42)	IA Only (n = 12)	FQ/IA (n = 54)	FQ & IA (n = 14)
Age groups				
< 35 years	64.3 (48.0–78.4)	83.3 (51.6–97.9)	59.3 (45.0–72.4)	85.7 (57.2–98.2)
≥ 35 years	35.7 (21.6–52.0)	16.7 (2.1–48.4)	40.7 (27.6–55.0)	14.3 (1.8–42.8)
Gender				
Female	23.8 (12.1–39.4)	25.0 (5.5–57.4)	11.1 (4.2–22.6)	35.7 (12.8–64.9)
Male	76.2 (60.5–87.9)	75.0 (42.8–94.5)	88.9 (77.4–95.8)	64.3 (35.1–87.2)
History of TB treatment				
Previously treated	64.3 (48.0–78.4)	66.7 (34.9–90.1)	59.3 (45.0–72.4)	71.4 (41.9–91.6)
New case	33.3 (19.6–49.5)	33.3 (10.0–65.1)	38.9 (25.9–53.1)	28.6 (8.4–58.1)
Treatment history unknown	0 (0.0)	0 (0.0)	0 (0.0)	0 (0.0)
Not provided	2.4 (0.1–12.6)	0 (0.0)	1.9 (0.0–9.9)	0 (0.0)

*N* Sample size, *n* Frequency, *Rx* Regimen, *%* Percentage, *FQ* Fluoroquinolone, *IA* Injectable agent, FQ Only=Pre−XDR

**Fig. 2:**
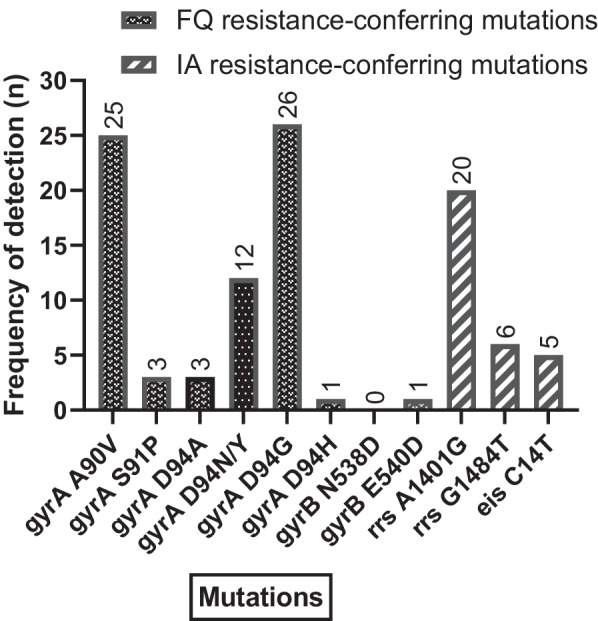
Distribution of gene mutations among second-line drug resistant tuberculosis patients; June 2017-December 2019

Eleven types of SLD resistance-conferring mutations in 4 genes (including the *eis* gene) were screened for in all 576 patients. Overall, 102 resistance-conferring mutations were identified in the samples tested, of which 70/102 (68.6%) conferred resistance to FQ, the majority in the *gyrA* locus, and 1/102 (0.98%) in the *gyrB* locus. The mutations D94G 42.6% (30.0–55.9) and A90V 41.1% (28.6–54.3) contributed the highest to *gyrA* locus proportions among the DR-TB patients. A total of 26 of the other mutations conferred resistance to IA, whereas 5 were associated with low-level resistance to kanamycin (KAN). On the other hand, 20/26 77.0% (56.4–91.0) of mutations conferring drug resistance to IA were observed for the *rrs* gene by the A1401G mutation 77.0% (56.4–91.0), Fig. 2*.*

Resistance profiles to SLD were distributed differently among patients. Four patients were found to have low-level drug resistance conferring mutations in the *eis* locus, independent of other mutations known to confer drug-resistance to any of FQ or IA. The pattern of SLD resistance-conferring mutations is presented in Table 4.

**Table 4:** Drug resistance-conferring mutation profiles among TB patients with second line drug-resistance

Drug (N = 68)	Resistance conferring mutation profile (s)	n (%; 95% CI)
FQ (Pre-XDR)		
	*gyrA* (A90V)	11 (16.2; 8.4–27.1)
	*gyrA* S91P	2 (2.9; 0.4–10.2)
	*gyrA* (D94A)	1 (1.5; 0.0–7.9)
	*gyrA* (D94N/Y)	8 (11.8; 5.2–21.8)
	*gyrA* (D94G)	13 (19.1; 10.6–30.5)
	*gyrA* (A90V, D94G)	3 (4.4; 0.9–12.4)
	*gyrA* (A90V, D94N/Y, D94G)	4 (5.9; 1.6–14.4)
IA		
	*rrs* (A1401G)	6 (8.8; 3.3–18.2)
	*rrs* (G1484T)	6 (8.8; 3.3–18.2)
FQ & IA		
	*gyrA* (A90V)/*rrs* (A1401G)	7 (10.3; 4.2–20.1)
	*gyrA* (D94G)/*rrs* (A1401G)	1 (1.5; 0.0–7.9)
	*gyrA* (D94G)/*rrs* (A1401G)	3 (4.4; 0.9–12.4)
	*gyrA* (S91P, D94A/*rrs* (A1401G)	1 (1.5; 0.0–7.9)
	*gyrA* (D94A, D94G)/*rrs* (A1401G)	1 (1.5; 0.0–7.9)
	*gyrA* (D94H, D94G)/*gyrB* (E540D)/rrs (A1401G)/*eis* (C14T)	1 (1.5; 0.0–7.9)

*N* Sample size, *n* Frequency, *%* Percentage, *CI* Confidence Interval, *FQ* Fluoroquinolones, *IA* Injectable agents

## Discussion

In this study among RIF and/or INH- resistant TB patients, the prevalence of resistance to second line drugs was 9.3% for FQ, 4.5% for IA and 2.4% for both FQ and IA. Among the four genes we considered to describe the molecular characterization of second-line drug resistance of which D94G in the *gyrA* gene for FQ and A1401G in the *rrs* gene for IA were the most prevalent resistance-conferring mutations to SLD. Resistance to FQ was conferred by mutations in the QRDR documented by previous studies [16–18].

As previously documented [19], the majority 72.6% of patients in our study who were resistant to RIF and/or INH were previously treated for TB. In addition to this, our study further established that patients under the age of < 35 were the majority, in consistency with a study by Kirenga et al*.,* (2015)[20]. The low frequency of any resistance to RIF and/or INH 576/20,508 (2.8%) among the TB patients in our study may be due to selection bias as a result of the purposive nature of sampling for this study. It is worth noting that this low proportion of drug-resistance is within the low Sub-Saharan estimates [21, 22] and also given the low DR-TB burden in our setting. This may be attributed to the strong directly-observed treatment strategy mechanisms and regulations against an over-the-counter sale of Anti-TB drugs in Uganda. All these combined efforts work in synergy to influence patient care by strengthening laboratory systems, eventually increasing access to DST and reduced anti-TB drug misuse to improve TB patient management.

The high number of samples subjected to DST in our setting may be attributed to the fact that the NTRL serves as a public TB culture laboratory, with a functional hub transport system to link specimens for further testing [23].

Mono-resistance to RIF was higher than INH monoresistance and is backed by recent studies that have raised more awareness on this phenomenon [24, 25]. The low proportions of INH mono-resistance at 9.8% (4.8–17.3) in this study may be attributed to a selection bias as a result of our selected study population. However, the higher proportions of RIF mono-resistance at 90.2% (82.7–95.2) are in agreement with two studies that raise awareness of increasing levels of this type of drug resistance among TB patients [24, 25]. Malenfant and Brewer [24] recently reported that the availability of diagnostic assays that allow for quick detection of RIF resistance has increased awareness of patients with rifampicin mono-resistant TB (RR-TB), which was previously thought to be uncommon [24]. A study conducted in South-western Uganda by Micheni et al*.* [25] also reported that the overall prevalence of monoresistance to INH and RIF was 8.5% and 11% respectively, while the prevalence of MDR-TB was 6.7% [25].

We observed a consistent proportion of multidrug-resistance among DR-TB patients, of whom, majority were RR-TB on the GeneXpert. This is in agreement with the global statistics of an estimated proportion of 82% living with MDR-TB among RR-TB patients. This is also still within range of the estimated 78% proportion of RR-TB patients that had MDR-TB as disclosed by the United Nations General Assembly (2018) on the fight against TB disease [1, 2]. The higher number of RIF resistant cases; 566 (79.8%) is attributed to the robust Xpert network in the country for detection of RR-TB patients, whose samples are thereafter referred for DST beyond RIF at the NTRL.

Our study findings also revealed a key establishment of a low proportion 68/576 (11.8%) of SLD resistance in which 9.3% and 4.5% were classified with at least resistance to FQ and IA respectively. This relatively low proportion of SLD resistance may be due to the sampling since a study of DR-TB prevalence requires other designs, with additional testing methods. Furthermore, the low SLD resistance proportion may be explained by previous documentation that drug resistance beyond first line anti-TB drugs and compensatory mutations remain low among TB patients in Uganda [26]. According to previous documentation by Theron et al*.* [27], the Hain Genotype MTBDR*sl* assay misses about one out of every five instances of FQ-resistant TB, as well as one out of every four cases of IA-resistant TB [27]. Nevertheless, the overall FQ resistance rate being lower than the global average of 21% [4] is quite encouraging. It especially calls for high regard of timely universal DST using points of care tests covering second line and novel anti-TB drugs to ascertain these estimates or even keep the numbers low and manageable if found to be accurate.

Given the fact that SLD resistance testing utilizing the Hain Genotype MTBDR*sl* assay was only available at the National Reference Level during our study period, we cannot rule out the likelihood that drug resistance to FQ and/or IA is higher than what we have reported.

Consistent with studies done elsewhere, among patients resistant to FQ in our study, resistance was mostly conferred by the *gyrA* [16, 28, 29] locus whereas resistance to second-line IA was mainly conferred by the *rrs* gene at codon A1401G [30]. Additionally, five *eis* C14T mutations were detected in the *eis* gene locus and led to low-level resistance to KAN [31], of which four occurred independent of mutations in the *rrs* gene. This type of mutations in the *eis* promoter region are suggestive of low-level KAN resistance as widely reported [32–34] but AMK remains effective [35]. Mutations in this region have been found to be associated with phenotypic KAN resistance in TB irrespective of whether the studied strains did or did not contain any mutations in the promoter region [36, 37].

The reported higher proportion of SLD resistant patients with resistance to any of FQ or IA as compared to those resistant to both FQ and IA is consistent with findings from recent global TB reports [1, 2, 4]. Our findings show a link between age and IA resistance as well as FQ and IA resistance, with patients younger than 35 years old being more susceptible to both types of SLD resistance. Like studies conducted elsewhere, gender was found to be a more susceptible determinant for SLD resistance. Males were more likely than females to develop FQ/IA resistance. Similar to findings from a prospective study that involved eight countries [38], males were more susceptible to develop FQ resistance than females. However, the study only included South Africa, a high-burden setting, as the only African country among the eight. In our study, the higher frequency of drug-resistance to FQ, IA, FQ/IA and FQ & IA among < 35-year-olds, previously treated patients and males may be attributed to the respective dominance of proportions of male participants.

One interesting observation from our study is the way in which the highest frequency (68.63%) of mutations known to confer drug resistance to FQ was observed in the *gyrA* gene locus in particular, the D94G (37.14%) and A90V (35.71%) mutations. Only one mutation, E540D, was detected in the *gyrB* locus among SLD resistant patients. This is not uncommon, given the fact that a rare occurrence has been cited in this locus [39]. This documentation, together previous studies that highlighted a rare occurrence of mutations in the *gyrB* gene locus [39] as well as a discrepancy in the ability to detect some mutations using some of the most commonly employed molecular diagnostics [16] may partly explain the under-representation of mutations in this gene locus among other reasons. Varying sensitivity levels have recently been reported especially with the detection of FQ for the tests performed using the WHO endorsed Genotype MTBDRsl v2.0 kit [27].

The high-level resistance conferring mutations from the *gyrA* locus were more frequently observed among the DR-TB patients with FQ resistance only. Resistance to IA among the studied patients was mainly conferred by the *rrs* A1401G mutation 77.0% (56.4–91.0) and is consistent with other relevant reports [31]. Our finding is in agreement with literature pointing to this mutation being the most common molecular mechanism of drug resistance to the injectables-kanamycin and amikacin [31]. The *gyrA* D94N/Y, and *gyrA* D94G mutations, which are all linked to high-level resistance to moxifloxacin, a key FQ drug, were found to be among the top three most commonly observed mutations in the *gyrA* locus among DR-TB patients studied. When the *gyrA* D94G mutation is present neither LFX nor MFX are effective options for treatment, whereas the presence of A90V implies that high doses of MFX could be effective, as this mutation is associated with low-resistance to the drug. This underlines the need to highly regard timely surveillance and DST in order to protect the SLDs and emergence of pre-extensively drug-resistant (XDR) TB.

The higher frequency of DST results with relativity to other results points to the reality that LPA indeterminate results from this study were easily resolved from repeat testing. LPA indeterminate results have previously been reported to occur in 1.4–19.2% of TB specimens, depending on the smear- and culture-status of the tested samples, as well as sample type (extra pulmonary or pulmonary) [40].

Only one case of invalid results was recorded during our study period. This may be due to the fact that an invalid result nullifies the entire strip, as it accommodates both FQ and IA target gene loci. Invalid results have previously been reported to occur in only 6% of smear positive sputum samples upon repeat testing [41]. Such errors arise as a result of errors made during run setup, presence of amplification inhibitors and/or performance of the amplification reaction. The findings of our study were produced by highly experienced professionals who adhere to high standards of work and undergo regular capacity building.

Our study had no cases of inferred resistance, a phenomenon that depicts the conserved genetic nature of strains tested at the NTRL from the referring sites, and therefore demonstrates the relevance of the LPA in our local setting. Although these instances of inferred resistance can be common locally [42], they are uncommon globally (< 1% of isolates), with synonymous and non-synonymous mutations such as phylogenetic mutations reported to cause systematic cases like these.

All in all, increased uptake of SLD resistance detection may therefore prove to be helpful in the rapid detection of SLD drug resistance in settings with low burden such as ours, as efforts move to an all-oral regimen. This is mostly desirable with the introduction of suitable rapid molecular diagnostics that would contribute a lot in terms of protecting the all-oral regimen.

Our findings are based on a couple of strengths. In the first place, our study was done at the Uganda NTRL, a World Health Organisation (WHO) Supranational Reference Laboratory with high competency and skilled personnel, which assured the quality of the drug resistance results presented. Our study findings are based on a large sample size, which assures the statistical power for our conclusions. We ensured that expertise was employed as the basis for selecting a sample that was most useful to the purposes of the research. This was done with consideration of the eligibility criteria where quality assured phenotypic DST results and agreeing data for the assays were used. Purposive efforts were also put in place to make the sampling as representative of the population as possible. The laboratory information systems in place reduced human error and guided the inclusion criteria for SLD resistant samples.

However, our study has some limitations; the study involved a retrospective review of the available data, which may have suffered from some missing information. The suboptimal sensitivity of the Genotype MTBDR*sl* Version 2.0 assay is a limitation in our study, highlighting the need for target sequencing as a reference standard at national reference level. We also recognize the risk of selection bias associated with the sampling of the patients. The specific mutations for 10 patients could not be re-interpreted because the bands on the line probe assay strips had faded beyond interpretation, thereby reducing the number of those suitable for inclusion in the study. Their characterisation could not be achieved, since these were not captured in the Laboratory Information Systems, highlighting the need for laboratories to enter these characterisation data in a standardised manner for future use in translational research and guiding policy. Finally, our data presentations fall short of the recent WHO definition of XDR-TB [43], a short fall that limited our establishment of patients that would fit in this category. This stresses the need to remain vigilant about the emergence of XDR-TB among well documented high-risk TB patients.

## Conclusion

We document that among the second line drug resistant patients exists a high frequency of high-level resistance to fluoroquinolones, a pivotal category of second line anti-TB medicines. The emergence of high-level resistance to moxifloxacin among the patients resistant to SLD generally highlights the need for routine diagnostic and surveillance mechanisms to keep record of the specific mutations from the routinely employed molecular diagnostics and serve the aforementioned purposes. Utilizing the routinely generated laboratory data from rapid molecular diagnostic tools, and targeted sequencing may prove to be a valuable strategy for TB control in anticipation of drug resistance surveys in resource-limited settings.

## Data Availability

The datasets used and/or analysed during the current study are available from the corresponding author on reasonable request.

## Abbreviations

DNA: Deoxyribonucleic acid
DST: Drug susceptibility testing
LPA: Line probe assay
MDR-TB: Multidrug resistant tuberculosis
NTRL: National tuberculosis reference laboratory
QRDR: Quinolone resistance determining region
RR-TB: Rifampicin resistant tuberculosis
SLD: Second-line drug
TB: Tuberculosis
WHO: World Health Organisation
XDR-TB: Extensively drug resistant tuberculosis
